# Analysis of Dynamic Magnetoelastic Coupling in Mechanically Driven Magnetoelectric Antennas

**DOI:** 10.3390/s22020455

**Published:** 2022-01-07

**Authors:** Kevin Q. T. Luong, Yuanxun (Ethan) Wang

**Affiliations:** Electrical and Computer Engineering Department, University of California, Los Angeles, CA 90095, USA; ywang@ee.ucla.edu

**Keywords:** magnetoelastic, magnetoelectric, magnetostriction, antenna, modeling

## Abstract

Mechanically driven magnetoelectric antennas are a promising new technology that enable a reduction in antenna size by many orders of magnitude, as compared to conventional antennas. The magnetoelastic coupling in these antennas, a phenomenon playing a direct role in determining performance, has been modeled using approaches that are severely lacking in both accuracy and tractability. In response to this problem, we take a physics-based approach to the analysis of magnetoelastic coupling. We find that certain directions of applied stress will maximize the coupling and we derive general expressions to quantify it. Our results are applied in comprehensive simulations that demonstrate the dynamic nature of the coupling as well as the impact of various operating conditions and material properties. Our work contributes analytical expressions and associated insight that can serve not only as guidelines for the design of mechanically driven magnetoelectric antennas, but also as stepping stones towards the development of more accurate models.

## 1. Introduction

**Figure 1:**
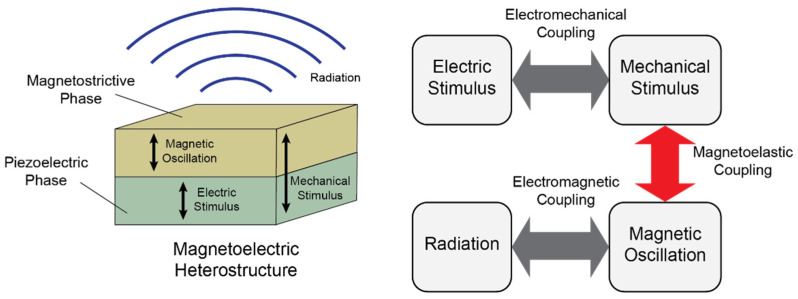
Heterostructure used for mechanically driven magnetoelectric antennas (**left**) along with operational flowchart (**right**) for electromagnetic wave radiation. Our work focuses specifically on an accurate analysis of the magnetoelastic coupling aspect of operation.

Magnetoelectric multiferroic materials exhibit both ferroelectricity and ferromagnetism and have a wide range of applications, from energy efficient memories to targeted drug delivery vehicles to photovoltaic devices [1]. A large appeal of these materials is their unique capability to enable electric field control of magnetism or vice versa. Recently, this capability has been taken advantage of to design what are referred to as mechanically driven magnetoelectric antennas [2]. These antennas employ multiferroic heterostructures composed of a piezoelectric phase and a magnetostrictive phase. Electromagnetic wave radiation is achieved by application of an electric voltage stimulus to the piezoelectric phase, which transduces the stimulus into a mechanical one. The mechanical stimulus then couples to the magnetostrictive phase, inducing oscillations of the magnetic moments that generate the radiation. For an oscillating magnetic dipole located in free space, centered at the origin of a coordinate system, and oriented in the $\hat{z}$ direction, its radiated far fields at a point $\left(r,\theta ,\phi \right)$ in spherical coordinates are given by:

(1) $$E=\frac{\mu_{0}m_{0}\omega^{2}}{4\pi c}\left(\frac{\sin\theta }{r}\right)\cos\left[\omega \left(t-r/c\right)\right]\hat{\phi }$$

(2) $$H=-\frac{m_{0}\omega^{2}}{4\pi c^{2}}\left(\frac{\sin\theta }{r}\right)\cos\left[\omega \left(t-r/c\right)\right]\hat{\theta }$$

where E is the electric field, H is the magnetic field, $\mu_{0}$ is the permeability of free space, $m_{0}$ is the magnetic moment of the dipole, ω is the angular frequency of its oscillation, c is the speed of light in free space, and SI units apply to all values [3]. This operational concept of mechanically driven magnetoelectric antennas is depicted in Figure 1. Electromagnetic wave reception by the antennas is achieved in an analogous but reversed procedure. By mechanically driving the magnetic moments responsible for radiation, antenna resonance is dictated by mechanical wave resonance [2]. This contrasts with the situation for conventional antennas, in which antenna resonance is dictated by electromagnetic wave resonance [4]. Given that mechanical wave velocities are many orders of magnitude slower than electromagnetic wave velocities and wavelength λ is related to wave velocity v according to fλ=v, with f being the wave frequency, mechanically driven magnetoelectric antenna dimensions can be reduced by many orders of magnitude as compared to those of conventional antennas operating at the same frequencies [2]. Further contrast exists in the fact that the source of radiation for mechanically driven magnetoelectric antennas is oscillating magnetic moments controlled by an electric voltage stimulus whereas for conventional antennas, the source of radiation is oscillating electric currents [4]. This eliminates ohmic losses to a large extent and improves antenna efficiency [2]. This also allows operation of the antenna in the presence of a ground plane without the platform effect and storage of reactive energy that conventional antennas are subject to [5].

While there has been progress in the realization of mechanically driven magnetoelectric antennas [2,6], the approaches to modeling and understanding them have been largely inappropriate. Consequently, there are no concrete guidelines with regards to how the antennas should be designed or operated to maximize their radiation and reception performance. In particular, we identify magnetoelastic coupling in the magnetostrictive phase of the antenna to be in most need of scrutiny. This coupling, highlighted in Figure 1, has been shown to be crucial in determining antenna performance with larger amounts of coupling entailing stronger transduction between mechanical stimuli and magnetic oscillations leading to more efficient radiation [5].

Past approaches tend to neglect a consideration of magnetization dynamics in their treatment of magnetoelastic coupling. They describe magnetic behavior entirely through either linear [7,8] or nonlinear [2,9] frequency-independent constitutive equations. In reality, magnetic behavior is highly dependent on the time-varying nature of its excitation in ways that simply cannot be described by these equations. Furthermore, past approaches tend to neglect the significance of many of the operating conditions and magnetic material properties that influence magnetoelastic coupling. The use of constitutive equations accounts for these factors only implicitly, obscuring the nature of their influence. The significance of magnetization dynamics, operating conditions, and material properties on magnetoelastic coupling has been clearly demonstrated in studies involving magnetostrictive materials as well as magnetostrictive-piezoelectric heterostructures [10,11]. Recent attempts have been made to incorporate magnetization dynamics in the modeling of mechanically driven magnetoelectric antennas [12]; nevertheless, operating condition and material property effects are still largely hidden by constitutive equations. All these past approaches additionally share the fact that they derive antenna performance from numerical simulations. These simulations provide little tangible insight to guide antenna design and operation choices.

In this paper, we address these problems by analyzing magnetoelastic coupling in mechanically driven magnetoelectric antennas using the physical equation governing magnetization dynamics. This equation is not only accurate in its description of magnetic behavior, but also it allows us to explicitly account for the effects of operating conditions and material properties. From this equation, we derive analytical expressions that quantify the coupling. These expressions provide insight into the design and operation choices that will maximize antenna performance. Our approach focuses solely on magnetoelastic coupling and so, in contrast with past approaches [2,7,9,12], electrodynamics are not considered. In the following sections, we introduce the equations governing magnetization dynamics along with the operating conditions and material properties of interest. We quantify magnetoelastic coupling, then explore theoretical conditions for its maximization. We finally derive expressions for the coupling and evaluate them for a variety of operating conditions and materials.

## 2. Methods

The physics of macroscopic magnetization dynamics are governed by the Landau-Lifshitz-Gilbert (LLG) equation. This equation and its numerical evaluation are the primary focus of standard magnetic simulation software [13]. We pursue an analytical evaluation here, however, to gain insight into the nature of magnetoelastic coupling and the factors that influence it. The equation and our approach to analysis are described first. We then introduce the operating conditions and material properties of interest to mechanically driven magnetoelectric antennas as well as how these factors are incorporated into the analysis.

### 2.1. Magnetization Dynamics

The LLG equation describing magnetization dynamics is given by:

(3) $$\frac{\partial M}{\partial t}=-\gamma \left(M\times H\right)+\frac{\alpha }{M_{s}}\left(M\times \frac{\partial M}{\partial t}\right)$$

where M is the magnetization in emu/cm^3^ with saturation magnetization $M_{s}$, H is an effective magnetic field in Oe, α is a damping constant, and γ is the gyromagnetic ratio in rad/(sec·Oe) [14].

Given that mechanically driven magnetoelectric antennas radiate or receive electromagnetic waves through a perturbation of magnetization, linear analysis is suitable and employed throughout this work. The LLG equation can be linearized [15] by supposing that the magnetization and effective magnetic field can be decomposed into a dominant static component and a small time-varying perturbation:

(4) $$M=M_{s}\hat{z}+m\;H=H_{z}\hat{z}+h$$

where $M_{s}\gg \left|m\right|$ and $H_{z}\gg \left|h\right|$. The dominant static components are parallel, here arbitrarily taken to lie along the *z*-axis of the laboratory coordinate system.

Neglecting higher order powers of the time-varying perturbations and assuming the system to be $e^{j\omega t}$ time-harmonic, (3) can be written as:

(5) $$\frac{1}{\gamma M_{s}}\left[\begin{array}{ccc} \gamma H_{z}+j\omega \alpha & -j\omega & 0\\ j\omega & \gamma H_{z}+j\omega \alpha & 0\\ 0 & 0 & 1 \end{array}\right]\left[\begin{array}{c} m_{x}\\ m_{y}\\ m_{z} \end{array}\right]=\left[\begin{array}{c} h_{x}\\ h_{y}\\ 0 \end{array}\right]$$

where $m_{i}$ and $h_{i}$, $i\in \left\{x,y,z\right\}$ are the phasor components of the perturbations. Our analysis of magnetoelastic coupling follows from an evaluation of (5) with the appropriate operating conditions and material properties of interest accounted for.

### 2.2. Operating Conditions & Material Properties

Several operating conditions and material properties are of particular interest for mechanically driven magnetoelectric antennas, motivating their incorporation into the evaluation of (5). Amongst operating conditions, foremost are the applied magnetic biasing and the applied stress. Magnetic biasing is important to ensure deterministic uniform magnetization as well as to ensure (4) is maintained, and stress is a fundamental mechanism upon which magnetoelectric antenna operation is contingent. Amongst material properties, foremost are the crystal and shape anisotropies. Crystal anisotropy causes magnetic behavior to vary based on the crystallographic direction of magnetization. Shape anisotropy causes magnetic behavior to vary based on the geometrical direction of magnetization. Also of interest are the material properties of saturation magnetization and damping; however, these are already represented explicitly in (5).

Each of these operating conditions and properties is associated with an energy density. Magnetic biasing is associated with Zeeman energy, applied stress with magnetoelastic energy, crystal anisotropy with magnetocrystalline anisotropy energy, and shape anisotropy with demagnetization energy. The crystal structure of a magnetic material will affect the nature of both the magnetoelastic and magnetocrystalline anisotropy energies. Equations for these energy densities can be found in magnetic material textbooks [16].

### 2.3. Effective Magnetic Field

The means by which the operating conditions and material properties of interest are incorporated into the evaluation of (5) is through the effective magnetic field, where each condition or property is represented with an additive contribution to this field. The contributions can be derived from the associated energy densities according to:

(6) $$H=-\frac{\partial W}{\partial M}$$

where W is the energy density in erg/cm^3^ [15]. Equations for the contributions of interest are summarized in Table 1.

The Zeeman contribution to effective magnetic field is simply the applied magnetic biasing field $H_{app}$.

For the magnetoelastic contributions, σ is the applied stress in dyne/cm^2^, the λ’s are magnetostriction constants, and $\hat{u}$ is parallel to the direction of applied stress. The equations for these contributions are given with respect to the crystal coordinate systems. The cubic crystal Equation (7) defines the coordinate system axes to be aligned with <100> directions, and the hexagonal crystal Equation (8) defines the coordinate system $\hat{z}$ axis to be aligned with the c-axis. There is no general energy density equation for polycrystals, and consequently no general equation for effective field contribution. However, under the condition that the grains of a polycrystal either have no preferred orientation or exhibit isotropic magnetostriction, (7) can be applied with $\lambda_{100}=\lambda_{111}=\lambda_{p}$ [16]. In this case, $\lambda_{p}$ is found as an average of magnetostriction over all grain orientations.

For the magnetocrystalline anisotropy contributions, the Ks are anisotropy constants in erg/cm^3^. The equations for these contributions are given with respect to the crystal coordinate systems, specified akin to those of (7) and (8) for cubic and hexagonal crystal respectively. Again, there is no general equation for polycrystals, in which the effective field contribution will be dependent on grain orientations. If there is no preferred orientation, the material will exhibit no net crystal anisotropy [16].

For the demagnetization contribution, the Ns are demagnetization coefficients. We are interested in magnetic bodies with simple geometries such as spheres, thin rods, or thin films in which the coefficients are approximately or exactly constant. For these geometries, it will also hold that two of the three coefficients are approximately equal [17]. In (11), $N_{t}$ is the value of the two equal coefficients, $N_{u}$ is the value of the third coefficient, and $\hat{d}$ is the direction corresponding to the third coefficient in the laboratory coordinate system.

## 3. Results

The described framework under which magnetization dynamics, operating conditions, and material properties are collectively represented is applied to assess magnetoelastic coupling. Towards this goal, magnetoelastic coupling is first quantified by defining coupling coefficients. Maximization of the coupling through optimal conditions of applied stress is then explored. General analytical expressions for the coupling coefficients are presented, then specialized for particular scenarios. Lastly, simulations are performed by evaluating the magnetoelastic coupling coefficient expressions for a variety of operating conditions and materials.

### 3.1. Optimal Applied Stress

For the system of (5), magnetoelastic coupling is quantified by coupling coefficients η defined to be the phasor ratio of the magnetization perturbation m components to the scalar applied stress σ. The phasor *z*-component of the perturbation is seen to be zero, and so coupling is described by two coefficients:

(12) $$\eta_{x}=\frac{m_{x}}{\sigma }\;\eta_{y}=\frac{m_{y}}{\sigma }.$$

From (12), larger amounts of magnetoelastic coupling correspond to larger perturbations of magnetization for a given amount of applied stress. This is clearly desirable for mechanically driven magnetoelectric antennas from the standpoint of radiation performance.

Amongst the many potential parameters that can be optimized to maximize magnetoelastic coupling, we focus on the conditions of applied stress. Applied stress manifests in (5) as a contribution to the effective magnetic field. Heuristically, (12) is maximized with a stress that maximizes the components of this contribution transverse to the dominant static component of magnetization.

From Table 1, applied stress is seen to be associated with two parameters. The first is σ, the magnitude of which quantifies the amount of stress and the sign of which indicates whether the stress is compressive or tensile. The second is $\hat{u}$, which indicates the direction of stress application. Given that the system of (5) is linearized, the magnitude of σ will not affect the value of the coefficients of (12). Furthermore, the sign of σ is irrelevant, given that the system of (5) is assumed to be time-harmonic. Consequently, an optimal applied stress that maximizes magnetoelastic coupling is tantamount to an optimal direction of application $\hat{u}$. We find this optimal direction for both cubic and hexagonal crystals.

#### 3.1.1. Cubic Crystal <100>

For cubic crystals with easy axes along the <100> directions, it is reasonable to suppose that the dominant static component of magnetization will be aligned along one of these directions. Given the magnetization of (4), (7) can be employed directly since it defines the crystal coordinate system axes to be aligned with <100> directions.

Neglecting the time-varying perturbation m for the time being, (7) can be written as:

(13) $$H=\frac{3\sigma \lambda_{100}}{M_{s}}u_{z}^{2}\hat{z}+\frac{3\sigma \lambda_{111}}{M_{s}^{2}}u_{z}\left[M_{s}u_{x}\hat{x}+M_{s}u_{y}\hat{y}+\hat{z}\right].$$

The magnitude of the transverse component of (13) is:

(14) $$\left|H_{t}\right|=\left|\frac{3\sigma \lambda_{111}}{2M_{s}}\sin2\theta \right|$$

where θ is the angle between $\hat{u}$ and the direction of dominant static magnetization. Equation (14) indicates that the optimal applied stress is directed at an angle 45° from the direction of dominant static magnetization.

#### 3.1.2. Cubic Crystal <111>

For cubic crystals with easy axes along the <111> directions, it is reasonable to suppose that the dominant static component of magnetization will be aligned along one of these directions. Given the magnetization of (4) and the crystal coordinate system definition for (7), a transformation between laboratory and crystal coordinate systems is needed in order to find the optimal applied stress. The details of this transformation are provided in Appendix A. Hereon, the prime symbol will distinguish vectors and vector components of the crystal coordinate system from those of the laboratory coordinate system.

Neglecting the time-varying perturbation m for the time being, (7) can be written as:

(15) $$\begin{array}{cc} H= & \frac{3\sigma \lambda_{100}}{M_{s}\sqrt{3}}\left[u_{x}^{\prime }{}^{2}{\hat{x}}^{\prime }+u_{y}^{\prime }{}^{2}{\hat{y}}^{\prime }+u_{z}^{\prime }{}^{2}{\hat{z}}^{\prime }\right]\\ & +\frac{3\sigma \lambda_{111}}{M_{s}\sqrt{3}}\left[\left(u_{y}^{\prime }+u_{z}^{\prime }\right)u_{x}^{\prime }{\hat{x}}^{\prime }+\left(u_{x}^{\prime }+u_{z}^{\prime }\right)u_{y}^{\prime }{\hat{y}}^{\prime }+\left(u_{y}^{\prime }+u_{x}^{\prime }\right)u_{z}^{\prime }{\hat{z}}^{\prime }\right]. \end{array}$$

Transforming (15) as well as ${\hat{u}}^{\prime }$ to the laboratory coordinate system, the magnitude of the transverse component of the result is found to be:

(16) $$\begin{array}{c} \left|H_{t}\right|=\left|\frac{\sigma }{M_{s}\sqrt{2}}\right|\sqrt{{\left(2A_{1}u_{x}u_{y}+A_{2}u_{x}u_{z}\right)}^{2}+{\left(A_{1}u_{x}^{2}-A_{1}u_{y}^{2}+A_{2}u_{y}u_{z}\right)}^{2}}\\ A_{1}=\lambda_{111}-\lambda_{100}\\ A_{2}=\sqrt{2}\left(2\lambda_{100}+\lambda_{111}\right). \end{array}$$

Letting stress be applied in the xz-plane, (16) simplifies to:

(17) $$\left|H_{t}\right|{\left.\right|}_{u_{y}=0}=\left|\frac{\sigma \sin\theta }{M_{s}\sqrt{2}}\right|\sqrt{A_{1}^{2}\sin^{2}\theta +A_{2}^{2}\cos^{2}\theta }$$

and letting stress be applied in the yz-plane, (16) simplifies to

(18) $$\left|H_{t}\right|{\left.\right|}_{u_{x}=0}=\left|\frac{\sigma \sin\theta }{M_{s}\sqrt{2}}\left(A_{1}\sin\theta -A_{2}\cos\theta \right)\right|$$

where θ is the angle between $\hat{u}$ and the direction of dominant static magnetization. The optimal $\hat{u}$ that maximizes (16) in general depends on the magnetostriction constants of the material. It will be characterized by not only θ, but also the angle ϕ of $\hat{u}$ in the plane transverse to the direction of dominant static magnetization. Section 3.3 shows that, for a given ϕ, the optimal θ will be approximately 45°, 90°, or 135° depending on the specific magnetic material.

#### 3.1.3. Hexagonal Crystal

For hexagonal crystals with easy axis along the c-axis, it is reasonable to suppose that the dominant component of magnetization will be aligned along that direction. Given (4), (8) can be employed directly, since it defines the $\hat{z}$ axis to be aligned with crystal c-axis.

Neglecting the time-varying perturbation m for the time being, (8) can be written as:

(19) $$H=\left(-\lambda_{A}-\lambda_{c}+4\lambda_{D}\right)\frac{\sigma }{M_{s}}u_{z}\left(u_{x}\hat{x}+u_{y}\hat{y}\right)+\left(-\lambda_{B}+\lambda_{B}u_{z}^{2}-\lambda_{C}u_{z}^{2}\right)\frac{2\sigma }{M_{s}}\hat{z}.$$

The magnitude of the transverse component of (19) is:

(20) $$\left|H_{t}\right|=\left|\left(-\lambda_{A}-\lambda_{c}+4\lambda_{D}\right)\frac{\sigma }{2M_{s}}\sin2\theta \right|$$

where θ is the angle between $\hat{u}$ and the direction of dominant static magnetization. Similar to the case of cubic crystals with dominant magnetization along a <100> direction, (20) indicates that the optimal applied stress is directed at an angle 45° from the direction of dominant static magnetization.

#### 3.1.4. Polycrystalline Material

For polycrystalline materials, the lack of a general equation describing the magnetoelastic contribution to the effective magnetic field implies the lack of a general equation to find optimal applied stress. Nevertheless, under the condition that the grains of a polycrystal either have no preferred orientation or exhibit isotropic magnetostriction, either (14) or (16) apply with $\lambda_{100}=\lambda_{111}=\lambda_{p}$. The optimal applied stress is then directed at an angle 45° from the direction of dominant static magnetization. This result is consistent with experimental demonstrations [10].

### 3.2. Magnetoelastic Coupling

Magnetoelastic coupling is quantified by the coupling coefficients (12). As they stand however, the equations of (12) are not very helpful in understanding the nature of magnetoelastic coupling and the factors that influence it. Expressions for these coefficients in terms of operating conditions and material properties of interest are found by solving (5) with the appropriate contributions accounted for in the effective magnetic field components $h_{x}$, $h_{y}$, and $H_{z}$. The contributions to these components are found through a linear analysis of the more general contributions of Table 1.

#### 3.2.1. Linearized Effective Magnetic Field Contributions

Table 2 presents linearized contributions for an effective magnetic field. These results were obtained based on the equations of Table 1, magnetization defined according to (4), and several assumptions made based on scenarios of interest. Amongst these assumptions, applied stress is taken to be optimally directed where known to maximize magnetoelastic coupling. For cubic crystals with dominant magnetization aligned along a <100> direction or hexagonal crystals with dominant magnetization aligned along the c-axis, this optimal direction is known. For cubic crystals with dominant magnetization aligned along a <111> direction, this optimal direction is material dependent, so some directions that produce simple results were chosen. The direction along which dominant magnetization is aligned is denoted in the first column of Table 2 along with the direction of applied stress in the format $\left(\theta ,\phi \right)$, where $\hat{u}=\sin\theta \cos\phi \hat{x}+\sin\theta \sin\phi \hat{y}+\cos\theta \hat{z}$. There is no general equation for the magnetoelastic contribution of polycrystalline materials; however, under the condition that the grains either have no preferred orientation or exhibit isotropic magnetostriction, (22) applies with $\lambda_{100}=\lambda_{111}=\lambda_{p}$. Other assumptions include the magnetic biasing, which is taken to be directed along the dominant static component of magnetization, $H_{app}=H_{0}\hat{z}$. 

#### 3.2.2. General Solution

General expressions for the coefficients of (12) can be found by recognizing that, with the linearized contributions of Table 2, (5) can always be written in the form:

(30) $$\left[\begin{array}{ccc} B_{1} & -j\omega & 0\\ j\omega & B_{2} & 0\\ 0 & 0 & 1 \end{array}\right]\left[\begin{array}{c} m_{x}\\ m_{y}\\ m_{z} \end{array}\right]=\gamma M_{s}\sigma \left[\begin{array}{c} C_{1}\\ C_{2}\\ 0 \end{array}\right]\;B_{1}=\gamma H_{z}-\gamma M_{s}h_{x,other}/m_{x}+j\omega \alpha \;B_{2}=\gamma H_{z}-\gamma M_{s}h_{y,other}/m_{y}+j\omega \alpha \;C_{1}=h_{x,me}/\sigma \;C_{2}=h_{y,me}/\sigma$$

where $B_{1}$, $B_{2}$, $C_{1}$, and $C_{2}$ are constants. Here, $h_{x,me}$ and $h_{y,me}$ are used to denote specifically the magnetoelastic linearized effective magnetic field contributions (22)–(25), whereas $h_{x,other}$ and $h_{y,other}$ are used to denote all other contributions. Solving (30) for the magnetoelastic coupling coefficients yields:

(31) $$\eta_{x}=\gamma M_{s}\left(\frac{B_{2}C_{1}+j\omega C_{2}}{\omega_{r}^{2}-\omega^{2}}\right)\;\eta_{y}=\gamma M_{s}\left(\frac{B_{1}C_{2}-j\omega C_{1}}{\omega_{r}^{2}-\omega^{2}}\right)$$

(32) $$\omega_{r}=\sqrt{B_{1}B_{2}}.$$

These expressions describe the coefficients in their most general form. They can be seen to account for not only the frequency dependency of the coupling, but also the effects of operating conditions and material properties. For more utility, we specialize (31) and (32) for some specific scenarios. All scenarios will assume magnetic biasing according to (21) such that (4) can be maintained.

#### 3.2.3. Magnetic Biasing

Suppose magnetic biasing is the dominant contributor to effective magnetic field such that all other contributions, other than that due to applied stress of course, can be neglected. This may be the case for example with a polycrystal with no preferred grain orientation and approximately spherical shape, or simply a material for which $H_{0}$ is sufficiently large. In this case, (31) and (32) become:

(33) $$\eta_{x}=\gamma M_{s}\left(\frac{\omega_{r}C_{1}+j\omega C_{2}}{\omega_{r}^{2}-\omega^{2}}\right)\;\eta_{y}=\gamma M_{s}\left(\frac{\omega_{r}C_{2}-j\omega C_{1}}{\omega_{r}^{2}-\omega^{2}}\right)$$

(34) $$\omega_{r}=\gamma H_{0}+j\omega \alpha$$

where $C_{1}$ and $C_{2}$ depend on the applied stress, or in other words the type of crystal, its orientation with respect to the dominant magnetization component, and the direction of applied stress.

#### 3.2.4. Cubic Crystal Anisotropy

When cubic crystal anisotropy also contributes significantly to the effective magnetic field, (33) holds with:

(35) $$\omega_{r}=\gamma H_{0}+\gamma \frac{2K_{1}}{M_{s}}+j\omega \alpha$$

in the case where the dominant magnetization component is aligned along a <100> direction, or

(36) $$\omega_{r}=\gamma H_{0}-\gamma \frac{4K_{1}}{3M_{s}}+j\omega \alpha$$

in the case where the dominant magnetization component is aligned along a <111> direction. Consequently, the effect of cubic crystal anisotropy is equivalent to a change in the strength of magnetic biasing.

#### 3.2.5. Hexagonal Crystal Anisotropy

When hexagonal crystal anisotropy contributes significantly to the effective magnetic field, then (33) holds with (35) in the case where the dominant magnetization component is aligned along the c-axis. In this case, $K_{1}$ would be the anisotropy constant associated with hexagonal anisotropy. This indicates that the effect of hexagonal crystal anisotropy, like that of cubic crystal anisotropy, is effectively a change in the strength of magnetic biasing.

#### 3.2.6. Demagnetization

Supposing demagnetization contributes significantly to the effective magnetic field, and defining $\theta_{d}$ to be the angle between the vector $\hat{d}$ of (11) and the dominant static component of magnetization, then we consider the two cases of $\theta_{d}$ equal to 0 and 90 degrees. With $\theta_{d}$ equal to 0 degrees, then (33) holds with:

(37) $$\omega_{r}=\gamma H_{0}+\gamma \left(N_{t}-N_{u}\right)M_{s}+j\omega \alpha .$$

In other words, demagnetization in this case is equivalent to a change in the strength of magnetic biasing. With $\theta_{d}$ equal to 90 degrees, we let $\hat{d}=\hat{x}$. No loss in generality is incurred with this choice given that a coordinate transformation can always be made to satisfy it. The coupling coefficients are then found to be described by (31) and (32) with:

(38) $$B_{1}=\gamma H_{0}+\gamma M_{s}\left(N_{u}-N_{t}\right)+j\omega \alpha \;B_{2}=\gamma H_{0}+j\omega \alpha .$$

### 3.3. Simulation

Simulations are performed using the magnetoelastic coupling coefficient results of Section 3.2 to extract further insights regarding the nature of the coupling and the effects of the operating conditions and material properties of interest. Iron (Fe) is the magnetic material considered in these simulations, unless otherwise stated. Relevant characteristics of iron are provided in Appendix B. The material has easy axes along the <100> crystallographic directions, and so the dominant component of magnetization is assumed to be aligned along one of these directions.

**Figure 2:**
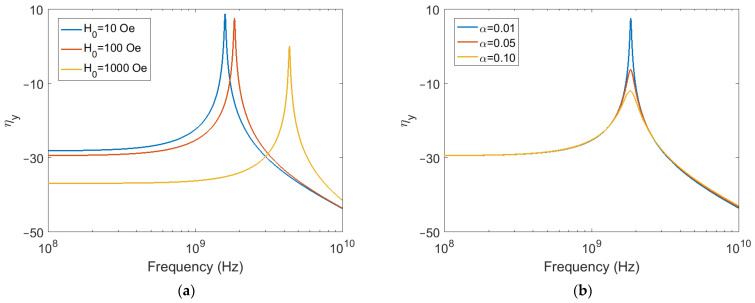
Magnetoelastic coupling coefficient magnitude for ϕ = 45°. (**a**) Varying magnetic biasing; (**b**) Varying magnetic damping.

The effects of magnetic biasing are demonstrated in Figure 2a, which is a plot of the magnitude of $\eta_{y}$ as a function of frequency of applied stress under various applied magnetic biasing field conditions. Given that our system is taken to be linear, this frequency is identical to the magnetization perturbation frequency. For the simulation, α is set to 0.01 and optimal stress is applied at an angle ϕ = 45° in the xy-plane, resulting in equal $\eta_{x}$ and $\eta_{y}$ magnitudes. Resonance behavior is exhibited, where the coupling is seen to reach a peak value at a particular frequency. It is also seen that stronger biasing fields raise the resonance frequency while reducing the amount of coupling both at and below resonance. Lastly, the magnetoelastic coupling coefficient for these results, as well as all other results presented in this section, displays asymptotic behavior for frequencies away from resonance.

The effects of damping are demonstrated in Figure 2b, which plots the magnitude of $\eta_{y}$ for various damping constant values. The biasing field for this simulation is set to 100 Oe and again, optimal stress is applied at an angle ϕ = 45° in the xy-plane, resulting in equal $\eta_{x}$ and $\eta_{y}$ magnitudes. It is seen that larger damping serves to lower the amount of coupling achieved at resonance, while having minimal effect on off-resonance coupling.

**Figure 3:**
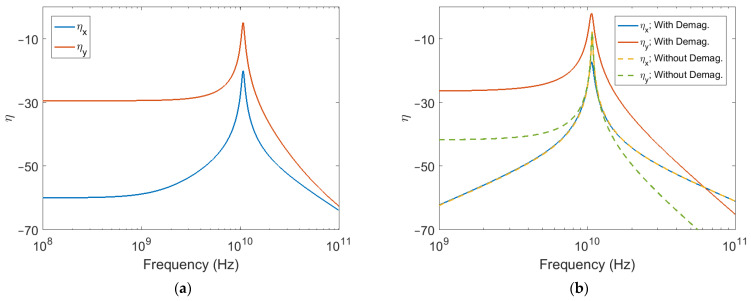
Magnetoelastic coupling coefficient magnitudes under the influence of thin film demagnetization with $\hat{d}=\hat{x}$. (**a**) Coefficients for ϕ = 45°; (**b**) Coefficients for ϕ = 90° compared to those of a material with no demagnetization and a bias field $H_{0}=3300$ Oe.

The effects of demagnetization are demonstrated in Figure 3. In Figure 3a, the magnitudes of both the $\eta_{x}$ and $\eta_{y}$ coupling coefficients are plotted as a function of frequency of applied stress for a thin film material geometry with $\theta_{d}$ = 90°, and specifically $\hat{d}=\hat{x}$, in which case $N_{t}\approx 0$ and $N_{u}\approx 4\pi$. The material is taken to have an α of 0.01, a biasing field of 100 Oe, and optimal stress applied at an angle ϕ = 45° in the xy-plane. Due to demagnetization, the magnetoelastic coupling exhibits anisotropy with respect to the angle of stress application in the xy-plane. In particular, the amount of coupling to the geometrical in-plane component of magnetization perturbation will tend to be much larger than that to the geometrical out-of-plane component. This is seen in Figure 3a both below and around resonance. Comparing with the analogous 100 Oe plot in Figure 2a, it is also seen that demagnetization acts to increase resonance frequency.

As we had seen in Figure 2a, a larger resonance frequency achieved by increasing the biasing field tends to lower the amount of coupling both at and below resonance. In contrast, a larger resonance frequency achieved due to demagnetization is not necessarily associated with the same drop in coupling. Figure 3b plots the magnitudes of the magnetoelastic coupling coefficients as a function of frequency of applied stress for the thin film material previously considered, now with ϕ = 90°, alongside those of the same material without any influence from demagnetization. The material without demagnetization is biased with a field of 3300 Oe to achieve the same resonance frequency as the thin film material. From the results, it is seen that the thin film material exhibits higher amounts of coupling to the *y*-component of magnetization perturbation at all frequencies. Coupling to the *x*-component of magnetization perturbation is the same amongst the two materials except at resonance, where the material without demagnetization reaches a higher value.

Iron was considered for all simulations so far. Other cubic crystal magnetic materials with dominant magnetization aligned along a <100> direction will have different saturation magnetizations, magnetocrystalline anisotropy constants, and magnetostriction constants. From the results of Section 3.2, these differences scale the amount of coupling or change the effective biasing field as compared to that of iron; however, the core behaviors demonstrated with iron still hold in general. For hexagonal materials with dominant magnetization aligned along the c-axis, again the amount of coupling may be scaled, or the effective biasing field changed, but the core behaviors remain the same. Likewise, only the specific demagnetization scenario of a thin film material with $\theta_{d}$ = 90° was considered so far. With other geometries or $\theta_{d}$ values, provided (21) is still satisfied, the effective biasing field, degree of anisotropy, and directions of anisotropy may change, but the core insights remain the same.

**Figure 4:**
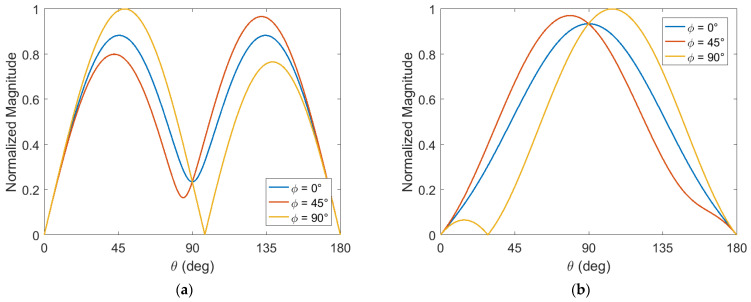
Normalized magnitude of the transverse effective magnetic field contribution from applied stress: (**a**) for nickel; (**b**) for magnetite.

On the other hand, cubic crystals with dominant magnetization aligned along a <111> direction will exhibit some fairly different behaviors. In particular, we found in Section 3.1 that the optimal direction of applied stress for these crystals in general depends not only on θ, but also ϕ, where $\hat{u}=\sin\theta \cos\phi \hat{x}+\sin\theta \sin\phi \hat{y}+\cos\theta \hat{z}$. Investigating this further, we plot the normalized value of (16) as a function of θ for several different ϕ values in Figure 4. Figure 4a shows the results for nickel (Ni), and Figure 4b shows the results for magnetite (Fe_3_O_4_), both cubic crystals with <111> easy axes along which dominant magnetization is assumed to be aligned. Relevant characteristics of these materials are given in Appendix B. It is seen that the optimal θ for a given ϕ is either approximately 45°, 90°, or 135°, depending on the material. This holds true in general for other applicable materials as well. It is also seen that the choice of ϕ influences the maximum possible magnitude of (16) that can be obtained. For both magnetite and nickel, ϕ = 90° is shown to enable a larger magnitude, as compared to the other values considered. 

## 4. Discussion

Mechanically driven magnetoelectric antennas are hardly the only application envisioned for piezoelectric–magnetostrictive heterostructures, and there have been many recent studies dedicated to an assessment of the magnetization dynamics in these heterostructures for other purposes [18]. For example, a substantial amount of research has been performed in this regard for high density, low power data storage applications [19]. However, these works were concerned with magnetic switching dynamics and stepped or pulsed mechanical stimuli [20] whereas our work deals with harmonic dynamics and stimuli. A substantial amount of research has also been performed assessing magnetization dynamics that are induced by surface acoustic wave mechanical stimuli for applications such as magnetic sensing [21] or spintronics [22]. However, these works tend to focus on obtaining numerical [22] or experimental [23] results. Until our study, analytical results focused on the maximization of magnetoelastic coupling as well as the effects of operating conditions and material properties on coupling had been absent.

Limitations of the presented results include the fact that they require several modifications to account for the effects of nanoscale magnetism that become significant as the dimensions of the magnetic material are reduced [24]. For example, thin films with thicknesses on the order of nanometers, also known as ultrathin films [25], have magnetizations and magnetostriction constants that may differ considerably from those of the corresponding bulk materials [17]. Surface anisotropy and epitaxial stress must also be considered for these films. Surface anisotropy can be modeled with a magnetocrystalline anisotropy contribution to the effective magnetic field found using (6) with energy density W given by [26]:

(39) $$W=K_{eff}\sin^{2}\left(\theta \right)\;K_{eff}=K_{v}+\frac{2K_{s}}{t}$$

where $K_{eff}$ and $K_{v}$ are the effective and volume anisotropy constants, respectively, in erg/cm^3^, $K_{s}$ is the surface anisotropy constant in erg/cm^2^, θ is the angle between the magnetization and the axis of magnetocrystalline anisotropy, and t is the thickness of the film in cm. Epitaxial stress can be handled with an additional magnetoelastic contribution term in the effective magnetic field [17]. Other magnetic nanostructures such as nanoparticles or nanowires exhibit their own unique properties that have spurred the development of a large variety of cutting-edge technologies [27,28,29], but their usefulness for mechanically driven magnetoelectric antennas is yet to be seen.

Another limitation of the presented results arises from the fact that our work focuses only on the magnetoelastic coupling in mechanically driven magnetoelectric antennas. Consequently, interpretations of the results are most appropriate in such a context. Our work does not consider the spatially dependent electrodynamics governed by Maxwell’s Equations [3]:

(40) $$\nabla \times E=-\frac{\partial B}{\partial t}\;\nabla \times H=J+\frac{\partial D}{\partial t}$$

where B is the magnetic flux density, H is the magnetic field, D is the electric displacement, and J is the volume current density. Our work further does not consider the spatially dependent elastodynamics governed by the acoustic field theory [30]

(41) $$S=\frac{1}{2}\nabla v+\frac{1}{2}{\left(\nabla v\right)}^{T}\;\nabla \cdot T=\rho \frac{\partial^{2}v}{\partial t^{2}}$$

where S is the strain tensor, v is the particle displacement, T is the stress tensor, and ρ is the equilibrium mass density. Lastly, our work does not consider the full coupling between all the various dynamics, described by the constitutive relations [12]

(42) $$B=\mu_{0}\left(H+M_{me}\left(d_{b}T\right)+M_{em}\right)\;S=s^{B}T+\mu_{0}d_{b}M$$

where $M_{me}$ is the magnetization directly induced by mechanical stimuli, $M_{em}$ is the magnetization induced by all other factors, and the total magnetization $M=M_{me}+M_{em}$. The variable $d_{b}$ is the effective piezomagnetic constant, and $s^{B}$ is the elastic compliance. In the context of our work, $M_{me}$ is found from (30) and $d_{b}$ is determined by $C_{1}$ and $C_{2}$ of (30), and T is determined by the applied stress variables σ and $\hat{u}$. These phenomena are ultimately necessary to rigorously account for processes such as system resonance, mechanical loss, or electromagnetic radiation and so far, have been collectively considered only in numerical approaches [12]. Nevertheless, our results provide considerable insight into characteristics of the crucial yet often overlooked magnetoelastic coupling aspect of antenna operation.

Looking to the future, mechanically driven magnetoelectric antennas are a promising new technology that have the potential to realize antennas with dimensions smaller than those of conventional antennas, a consequence of their dependence on mechanical resonance, and efficiencies higher than those of conventional antennas, a consequence of their independence from electric currents [2]. As with any new technology however, mechanically driven magnetoelectric antennas are still subject to challenges inhibiting their development [31]. One of these challenges is with respect to fabrication, where current methods produce materials with undesirable residual stresses and domain structures. Another challenge is with respect to structural integrity, where design approaches that maintain a high degree of structural robustness are currently limited. Another challenge is of course the lack of appropriate means to model and understand the antennas and specifically the magnetoelastic coupling component of their operation, which is what our work addresses.

## 5. Conclusions

In this work, we approached the analysis of magnetoelastic coupling by employing the LLG equation to accurately represent magnetization dynamics and finding effective magnetic field contributions for various operating conditions and material properties to account for their effects. We quantified the coupling by defining magnetoelastic coupling coefficients and derived conditions of applied stress to maximize it. These conditions were found to be consistent with results from experimental studies in literature. We lastly derived analytical expressions relating the coupling coefficients to parameters of interest and performed comprehensive simulations to assess the characteristics of these coefficients in the frequency domain. The analytical expressions and associated insights presented are intended to serve as approximate guidelines for antenna design and operation choices as well as model development in order to enable researchers to realize the full capabilities of mechanically driven magnetoelastic antennas.

## Figures and Tables

**Table 1:** Effective magnetic field contributions.

Contributor	Effective Magnetic Field H (Oe)	
Zeeman	$H_{app}$	
Magnetoelastic		
Cubic ^1^	$\frac{3\sigma \lambda_{100}}{M_{s}^{2}}\left(u_{x}^{2}M_{x}\hat{x}+u_{y}^{2}M_{y}\hat{y}+u_{z}^{2}M_{z}\hat{z}\right)\;+\frac{3\sigma \lambda_{111}}{M_{s}^{2}}\left[\left(M_{y}u_{y}+M_{z}u_{z}\right)u_{x}\hat{x}\;+\left(M_{x}u_{x}+M_{z}u_{z}\right)u_{y}\hat{y}+\left(M_{y}u_{y}+M_{x}u_{x}\right)u_{z}\hat{z}\right]$	(7)
Hexagonal ^2^	$H_{A}+H_{B}+H_{C}+H_{D}$ where $H_{A}=\frac{\sigma \lambda_{A}}{M_{s}^{2}}\left[\left(M_{x}u_{x}+M_{y}u_{y}\right)\left(2u_{x}\hat{x}+2u_{y}\hat{y}-u_{z}\hat{z}\right)\right]-\frac{\sigma \lambda_{A}}{M_{s}^{2}}M_{z}u_{z}\left[u_{x}\hat{x}+u_{y}\hat{y}\right]$ $H_{B}=-\frac{2\sigma \lambda_{B}}{M_{s}^{2}}\left[\left(M_{x}u_{x}+M_{y}u_{y}\right)u_{x}\hat{x}+\left(M_{x}u_{x}+M_{y}u_{y}\right)u_{y}\hat{y}+M_{z}\left(1-u_{z}^{2}\right)\hat{z}\right]$ $H_{C}=-\frac{\sigma \lambda_{C}}{M_{s}^{2}}u_{z}\left[M_{z}u_{x}\hat{x}+M_{z}u_{y}\hat{y}+\left(M_{x}u_{x}+M_{y}u_{y}+2M_{z}u_{z}\right)\hat{z}\right]$ $H_{D}=\frac{4\sigma \lambda_{D}}{M_{s}^{2}}u_{z}\left[M_{z}u_{x}\hat{x}+M_{z}u_{y}\hat{y}+\left(M_{x}u_{x}+M_{y}u_{y}\right)\hat{z}\right]$	(8)
Polycrystal	No general equation	
Magnetocrystalline Anisotropy		
Cubic ^3^	$-\frac{2K_{1}}{M_{s}^{2}}M+\frac{2K_{1}}{M_{s}^{4}}\left(M_{x}^{3}\hat{x}+M_{y}^{3}\hat{y}+M_{z}^{3}\hat{z}\right)$	(9)
Hexagonal ^3^	$\frac{2K_{1}}{M_{s}^{2}}\left(M\cdot \hat{z}\right)\hat{z}$	(10)
Polycrystal	No general equation	
Demagnetization	$\left(N_{t}-N_{u}\right)\left(M\cdot \hat{d}\right)\hat{d}$	(11)

^1^ Valid for crystals with easy axes along the <100> or <111> directions. ^2^ Valid for crystals with easy axis along the c-axis. ^3^ Higher order terms are neglected.

**Table 2:** Linearized effective magnetic field contributions.

Contributor	$h_{x}$	$h_{y}$	$H_{z}$	
Zeeman	0	0	$H_{0}$	(21)
Magnetoelastic				
Cubic <100>; $\left(45{}^{\circ},\phi \right)$	$\frac{3\sigma \lambda_{111}}{2M_{s}}\cos\phi$	$\frac{3\sigma \lambda_{111}}{2M_{s}}\sin\phi$	0	(22)
Cubic <111>; $\left(45{}^{\circ},-45{}^{\circ}\right)$	$\frac{3\sigma \lambda_{100}}{2M_{s}\sqrt{2}}$	$-\frac{\sigma \left(2\lambda_{100}+\lambda_{111}\right)}{2M_{s}\sqrt{2}}$	0	(23)
Cubic <111>; $\left(90{}^{\circ},0{}^{\circ}\right)$	0	$-\frac{\sigma \left(\lambda_{100}+\lambda_{111}\right)}{M_{s}\sqrt{2}}$	0	(24)
Hexagonal (c-axis); $\left(45{}^{\circ},\phi \right)$	$\left(-\lambda_{A}-\lambda_{C}+4\lambda_{D}\right)\frac{\sigma }{2M_{s}}\cos\phi$	$\left(-\lambda_{A}-\lambda_{C}+4\lambda_{D}\right)\frac{\sigma }{2M_{s}}\sin\phi$	0	(25)
Polycrystal	No general equation			
Magnetocrystallline Anisotropy				
Cubic ^1^ [100]	$-\frac{2K_{1}}{M_{s}^{2}}m_{x}$	$-\frac{2K_{1}}{M_{s}^{2}}m_{y}$	0	(26)
Cubic [111]	0	0	$-\frac{4K_{1}}{3M_{s}}$	(27)
Hexagonal	0	0	$\frac{2K_{1}}{M_{s}}$	(28)
Polycrystal	No general equation			
Demagnetization ^1^	$\left(N_{t}-N_{u}\right)\left(m_{x}d_{x}+m_{y}d_{y}\right)d_{x}$	$\left(N_{t}-N_{u}\right)\left(m_{x}d_{x}+m_{y}d_{y}\right)d_{y}$	$\left(N_{t}-N_{u}\right)M_{s}d_{z}^{2}$	(29)

^1^ Under magnetostatic limit; general case requires consideration of electrodynamics.

## Data Availability

Not applicable.

## Appendix A. Coordinate System Transformation

For a cubic crystal with the [111] direction aligned along the $\hat{z}$ axis of the laboratory coordinate system, a vector transformation from crystal to laboratory coordinate system is given by:

(A1) $$T=\left[\begin{array}{ccc} -1/\sqrt{2} & 1/\sqrt{2} & 0\\ -1/\sqrt{6} & -1/\sqrt{6} & 2/\sqrt{6}\\ 1/\sqrt{3} & 1/\sqrt{3} & 1/\sqrt{3} \end{array}\right]$$

with the transpose $T^{T}$ being the inverse transformation from the laboratory to crystal coordinate system [15].

## Appendix B. Magnetic Material Properties

Several magnetic materials and their associated properties are provided in Table A1 [16,17,32].

**Table A1:** Magnetic material properties.

Material	Saturation Magnetization (emu/cm^3^)	Crystal Anisotropy ^1^ (erg/cm^3^)	Magnetostriction (10^−6^)
Fe	1714	$4.8\times 10^{5}$	$\lambda_{100}=21$ $\lambda_{111}=-21$
Ni	484	$-5\times 10^{4}$	$\lambda_{100}=-46$ $\lambda_{111}=-24$
Fe_3_O_4_	480	$-1.1\times 10^{5}$	$\lambda_{100}=-20$ $\lambda_{111}=78$
Co	1297	$2\times 10^{6}$	$\lambda_{A}=-45$ $\lambda_{B}=-95$ $\lambda_{C}=110$

^1^ Higher order terms neglected.
